# Post-translational modifications of beta-amyloid modulate its effect on cell mechanical properties and influence cytoskeletal signaling cascades

**DOI:** 10.3389/fnmol.2024.1501874

**Published:** 2024-11-14

**Authors:** Kseniya B. Varshavskaya, Evgeny P. Barykin, Roman V. Timoshenko, Vasilii S. Kolmogorov, Alexander S. Erofeev, Petr V. Gorelkin, Vladimir A. Mitkevich, Alexander A. Makarov

**Affiliations:** ^1^Engelhardt Institute of Molecular Biology, Moscow, Russia; ^2^Research Laboratory of Biophysics, National University of Science and Technology “MISIS”, Moscow, Russia; ^3^Department of Chemistry, Lomonosov Moscow State University, Moscow, Russia

**Keywords:** Alzheimer’s disease, beta-amyloid, post-translational modifications, scanning ion-conductance microscopy, ROS, actin cytoskeleton, cofilin, protein kinases

## Abstract

Post-translational modifications of beta-amyloid (Aβ) play an important role in the pathogenesis of Alzheimer’s disease (AD). Aβ modifications such as Ser8 phosphorylation (pS8-Aβ_42_) and Asp7 isomerization (iso-Aβ_42_) can significantly alter the properties of Aβ and have been detected *in vivo*. One of the reasons for the different pathogenicity of Aβ isoforms may be the activation of different signaling cascades leading to changes in the mechanical properties of cells. In this paper, we used correlative scanning ion-conductance microscopy (SICM) and Pt-nanoelectrodes to compare the effects of Aβ isoforms on the Young’s modulus of SH-SY5Y cells and the level of ROS. It was found that unmodified Aβ_42_ resulted in the largest increase in cell Young’s modulus of all isoforms after 4 h of incubation, while pS8-Aβ_42_ induced the greatest increase in stiffness and ROS levels after 24 h of incubation. Analysis of signaling proteins involved in the regulation of the actin cytoskeleton showed that Aβ_42_, pS8-Aβ_42_ and iso-Aβ_42_ have different effects on cofilin, GSK3β, LIMK, ERK and p38. This indicates that post-translational modifications of Aβ modulate its effect on neuronal cells through the activation of various signaling cascades, which affects the mechanical properties of cells.

## 1 Introduction

Alzheimer’s disease (AD) is the most common cause of dementia worldwide (Anand et al., 2014). One of the hallmarks of AD is the formation of amyloid plaques in various parts of the brain, consisting mainly of beta-amyloid peptide (Aβ). Aβ is known to undergo various post-translational modifications that affect its aggregation, toxicity, enzymatic degradation, and interaction with protein partners (Barykin et al., 2017). Many studies note that the presence of Aβ_42_ alone is not enough for the development of pathology: the appearance of modified forms that change the action of Aβ_42_ serves as a trigger for the disease (Barykin et al., 2017). Some of these modifications include phosphorylation at serine 8 (pS8-Aβ_42_) and isomerization of aspartic acid 7 (iso-Aβ_42_). Aβ containing isomerized Asp7 was found in more than 50% of the Aβ molecules of amyloid plaques, as well as in soluble fraction (Mukherjee et al., 2021). This modification is more prone to aggregation than unmodified Aβ and has greater toxicity and resistance to degradation by enzymes (Kummer and Heneka, 2014; Gnoth et al., 2020). Another important modification of Aβ, phosphorylation of Ser8, influences the neurotoxicity of the peptide and has been found in the brains of AD patients and in the brains of transgenic animals modeling AD (Kummer and Heneka, 2014; Kumar et al., 2018). We have shown that iso-Aβ_42_ is a more potent inhibitor of α7 nAChR than the unmodified peptide (Barykin et al., 2019), and the inhibition of Na^+^/K^+^-ATPase by beta-amyloid is completely eliminated by phosphorylation of the peptide at Ser8 (Barykin et al., 2018). Thus, pS8-Aβ_42_ and iso-Aβ_42_ represent important isoforms whose properties are significantly different from unmodified Aβ.

The difference in the pathogenicity of beta-amyloid isoforms for neuronal cells may be due to their different effects on the mechanical properties of cells. The mechanical properties of cells are closely related to many important biological functions, such as adhesion, division, motility, differentiation and deformation, and are controlled mainly by the cytoskeleton (Luo et al., 2016). Aβ has been shown to induce changes in the cytoskeleton and mechanical properties of neuronal cells (Ungureanu et al., 2016; Kolmogorov V. S. et al., 2023), but whether the modified forms have the same properties has not been previously studied. Establishing differences in the properties and effects of beta-amyloid isoforms may be important for a complete study of the mechanism of AD development.

**Figure 1:**
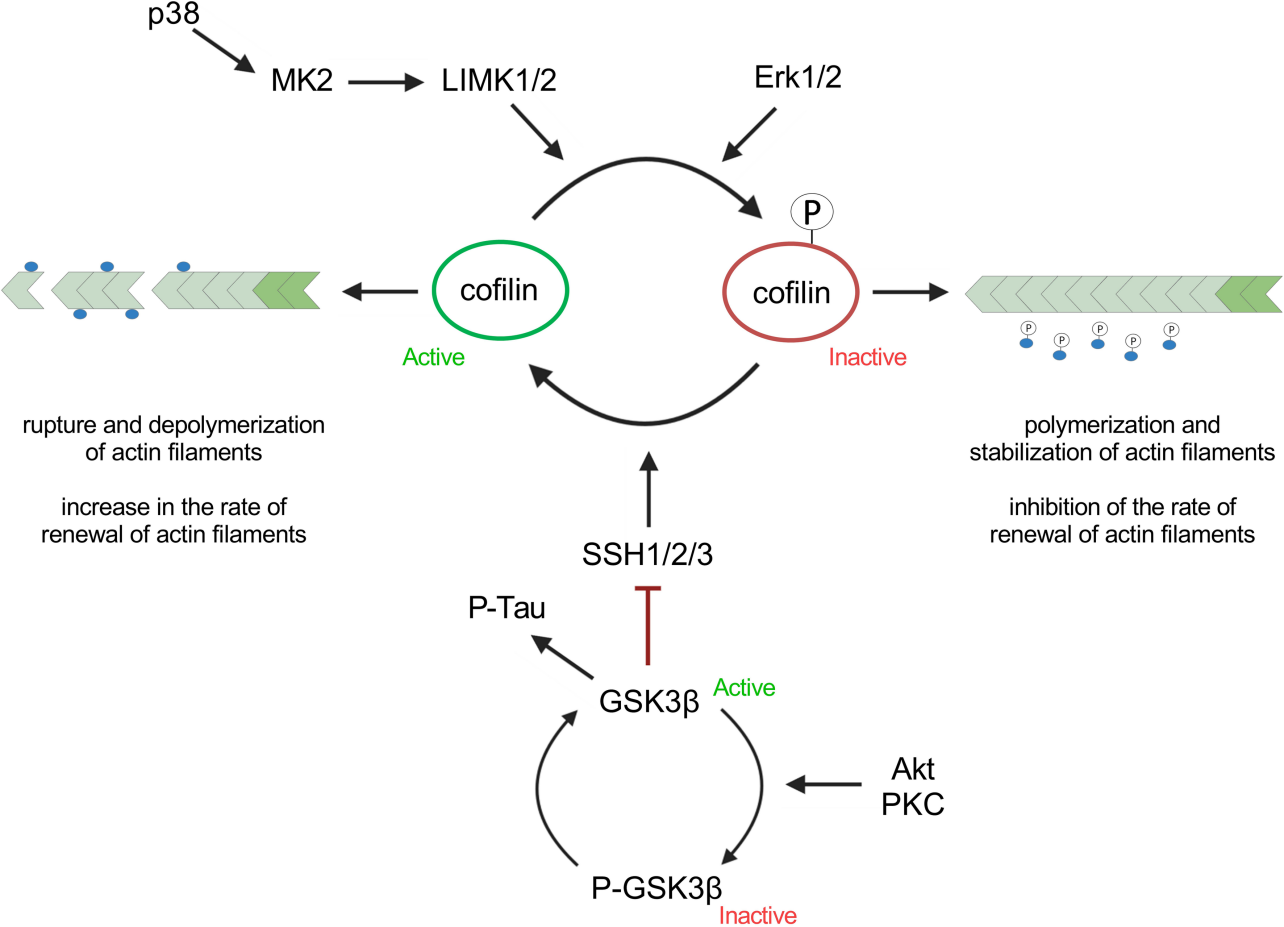
Signaling cascades involved in actin polymerization/depolymerization discussed in this article.

In this study, we measured cell stiffness and ROS production in response to Aβ_42_, pS8-Aβ_42_ and iso-Aβ_42_. Next, we aimed to discover which signaling cascades associated with the regulation of cell mechanical properties through influence on the actin cytoskeleton and ROS are activated by Aβ and its isoforms. Cofilin, which plays a key role in cytoskeletal dynamics, and its regulating kinases GSK3β, ERK1/2, p38 and LIMK1 were chosen as target proteins for studying the influence of Aβ isoforms (Figure 1).

## 2 Materials and methods

### 2.1 Preparation of synthetic beta-amyloid peptides

Synthetic peptides [H2N]-DAEFRHDSGYEVHHQKLVFFAEDVGSNKGAIIGLMVGGVVIA-[COOH] (Аβ_42_), [H2N]-DAEFRHD[pS]GYEVHHQKLVFFAEDVGSNKGAIIGLMVGGVVIA-[COOH] (pS8-Аβ_42_) and [H2N]-DAEFRH[isoD]SGYEVHHQKLVFFAEDVGSNKGAIIGLMVGGVVIA-[COOH] (iso-Аβ_42_) were obtained from Biopeptide (San Diego, CA, USA). Peptides were pre-monomerized by adding cold hexafluoroisopropanol (Fluka) to dry peptides to a concentration of 1 mM and incubating for 60 min at room temperature. The solutions were then aliquoted and dried in an Eppendorf 5,301 vacuum concentrator (Hamburg, Germany) for 20 min. The obtained dry peptides were stored at −20°C. Before the experiment, a 2.5 mM stock solution of Aβ_42_, pS8-Aβ_42_, or iso-Aβ_42_ was prepared by adding 10 μL of 100% anhydrous dimethyl sulfoxide (DMSO) (Sigma-Aldrich, St. Louis, MO, USA) to 0.12 mg of peptide, followed by incubation for 1 h at room temperature. The stock solution was then diluted to 10 μM using serum-free RPMI-1640 medium.

### 2.2 Cell culture

Experiments were performed on the human neuroblastoma cell line SH-SY5Y. Cells were cultured at 37°C in an atmosphere of 5% CO_2_ in RPMI-1640 medium (Gibco) containing 1% GlutaMax (Gibco), 100 U/mL penicillin and 100 μg/mL streptomycin (PenStrep; Gibco), with the addition of 10% fetal bovine serum (FBS). For experiments, SH-SY5Y cells were incubated with 10 μM Aβ_42_, pS8-Aβ_42_, or iso-Aβ_42_ or an equivalent amount of DMSO.

### 2.3 The amperometric detection of ROS using Pt-nanoelectrodes

The ROS concentration was determined by the amperometric method using Pt-nanoelectrodes. Fabrication of Pt-nanoelectrodes and single cell measurement process have been described in detail elsewhere (Erofeev et al., 2018; Vaneev et al., 2020).

Before experiments, SH-SY5Y (3 × 10^5^) were seeded in 35 mm Petri dish and treated after night with beta-amyloid peptides for 4 or 24 h. After the incubation time, attached cells in Petri dishes were washed three times by using Hanks’ Balanced Salt solution (HBSS) to remove the growth media and traces of amyloids. The total ROS level was calculated from the recorded intracellular average current value and calibration curve for each Pt-nanoelectrode.

### 2.4 Scanning ion-conductance microscopy

SICM by ICAPPIC (ICAPPIC ltd, United Kingdom) is used for topography and Young’s modulus mapping of SH-SY5Y cells. Nanopipettes with 45–50 nm in radius was made from borosilicate glass O.D. 1.2 mm, I.D. 0.69 mm (WPI, United Kingdom) by using laser puller P-2000 (Sutter Instruments, USA). Nanopipette radius was calculated by using by following model (Clarke et al., 2016):


$$r=\frac{\mathrm{Io}}{\mathrm{\pi Vktg}\left(\alpha \right)}$$

where the half-cone angle *α* is 3 degrees, *κ* is 1.35 S m^−1^ and V is the applied electrical potential of 200 mV.

A nanopipette was brought up close to the surface and scanned until the ion current through the tip decreased by 2% from its starting value in order to estimate the Young’s modulus of living cells (Kolmogorov et al., 2021). At an ion current decrease of 0.5%, a noncontact topographic image was obtained. Two more coordinates were obtained at ion current decreases (or set-points) of 1 and 2%, respectively, corresponding to membrane deformations induced by intrinsic force at each setpoint. Next, Young’s modulus was calculated using the model shown below:


$$E=PA{\left(\frac{\mathrm{Ssub}}{\mathrm{Scell}}-1\right)}^{-1}$$

where *E* is the estimated Young’s modulus, *P* is the applied pressure, A is a constant depending on the nanopipette geometry, and S_sub_ and S_cell_ are the slopes of the current–distance curve observed between the ion current decreases of 1 and 2% at the non-deformable surface (S_sub_ – substrate) and cell surface (S_cell_), respectively. Cells were washed several times with Hank’s solution (Gibco, USA) before scanning procedure. Cell density can influence Young’s modulus (Stroka and Aranda-Espinoza, 2011; Nehls et al., 2019), and we confirmed this in our model (Supplementary Figure S1). To avoid such effect, all experiments with cell stiffness were performed using the same seeding density.

### 2.5 Preparation of cell lysates to determine the degree of phosphorylation of signaling proteins

To determine the degree of phosphorylation of signaling proteins regulating the cytoskeleton, SH-SY5Y cells were seeded into wells of a 12-well plate (Greiner Bio-One) at 150 thousand per well and grown in RPMI-1640 medium with 10% FBS for 4 days. Before the experiment, the cells were washed with serum-free RPMI-1640 medium (500 μL per well), after which RPMI-1640 medium containing 10 μM Aβ_42_, pS8-Aβ_42_, iso-Aβ_42_ or an equivalent amount of DMSO (control solution) was added (450 μL per well) and incubated at 37°C in a CO_2_ incubator (Eppendorf) for 30 min, 2 or 4 h. After the incubation time, the cells were washed 2 times with PBS (Gibco) at 500 μL per well, the plate was cooled without liquid on ice for 5 min, then frozen in liquid nitrogen and placed in a kelvinator (−80°C) overnight. After that, 300 μL of lysis buffer (Millipore) with the addition of a protease inhibitor (Roche) and a phosphatase inhibitor (Thermo Fisher Scientific) were added to the wells and incubated for 15 min on ice. The cells were removed using a scraper, placed in 1.5 mL test tubes and incubated for 1 h at +4°С with stirring. The cell lysate was centrifuged at 16,000 g, +4°C, 10 min (Eppendorf centrifuge, 5415R) and the supernatant was collected. The amount of total protein in the lysates was determined using a BCA assay kit (Sigma) according to the manufacturer’s protocol.

### 2.6 Western blot

The cell lysate was mixed with Tris-glycine sample buffer (Bio-Rad) containing 5% beta-mercaptoethanol and heated in a solid-state thermostat (bioSan, TDB-120) at 95°C for 5 min. Proteins were separated in a 10% Tris-glycine polyacrylamide gel by Laemmli electrophoresis. Proteins were transferred using a semi-dry method (TransBlot Turbo, Bio-Rad) onto a 0.2 μm nitrocellulose membrane (Bio-Rad) and blocked in a 5% skim milk solution (Diaem) in TBST (50 mM Tis-HCl, pH 7.4, 150 mM NaCl, 0.1% Tween-20) for 1 h with stirring. The membranes were incubated with primary antibodies to the following proteins: (1) Cofilin (Cell Signaling Technology, D3F9, Rabbit, 5,175); (2) Phospho-Cofilin (Ser3) (Cell Signaling Technology, 77G2, Rabbit, 3,313); (3) LIMK1 (Cell Signaling Technology, Rabbit, 3,842); (4) Phospho-LIMK1 (Thr508)/LIMK2 (Thr505) (Cell Signaling Technology, Rabbit, 3,841); (5) GSK-3β (Cell Signaling Technology, D5C5Z, Rabbit, 12,456); (6) Phospho-GSK-3β (Ser9) (Cell Signaling Technology, D85E12, Rabbit, 5,558); beta actin (Abcam, ab8227). The membranes were incubated with primary antibodies overnight at +4°C with stirring, then washed in TBST and incubated with secondary antibodies to rabbit immunoglobulins conjugated with HRP (Hytest, 1:4,000) for 1 h at room temperature with stirring. Membranes were visualized on a Bio-Rad ChemiDoc MP using Supersignal West Pico PLUS chemiluminescent substrate (ThermoFisher Scientific). Quantitative analysis was performed using Image Lab 6.0.1 software. Original blot images are presented in Supplementary material. To determine changes in the total level of kinases and cofilin, we normalized the total level of proteins of interest to the total protein content in the sample. The total protein content was calculated as follows: (sample volume loaded on а gel) * (protein concentration in the lysate measured by BCA) * (dilution with loading buffer). Normalization by the levels of cytoskeletal proteins was not used because their levels changed under conditions of the experiment.

### 2.7 Milliplex

Lysate samples were diluted 2-fold in Assay Buffer (Milliplex, Millipore) and the degree of kinase phosphorylation was determined using Milliplex kits measuring the total form (MILLIPLEX MAP Multi-Pathway Total Magnetic Bead 9-Plex, 48-681MAG, Millipore) or the phosphorylated form of ERK and p38 kinases (MILLIPLEX Multi-Pathway Magnetic Bead 9-Plex, 48-680MAG, Millipore) according to the manufacturer’s protocol. Fluorescence intensity was measured on a MagPix instrument (Millipore) using calibration kits (MAGPIX Calibration Kit, Cat. No. MPX-CAL-K25), verification kits (MAGPIX Performance Verification Kit, Cat. No. MPX-PVER-K25) and xPONENT software, 4.3.229.0. Data processing was carried out using Belysa software v1.1.0 (Merck, Rahway, NJ, USA) at the Resource Center “Cell Technology and Immunology,” Sirius University of Science and Technology.

### 2.8 Statistical data processing

Experimental data are presented as the mean of independent experiments ± standard deviation (sd). The number of independent experiments is indicated in the figure legends. Statistical differences between experimental groups were determined using one-way ANOVA with Tukey’s test for multiple comparisons. Differences were considered statistically significant at *p* < 0.05. Statistical analysis was performed using GraphPad Prism 8.0.1 software.

## 3 Results

### 3.1 Effect of beta-amyloid isoforms on Young’s modulus and ROS levels in SH-SY5Y

**Figure 2:**
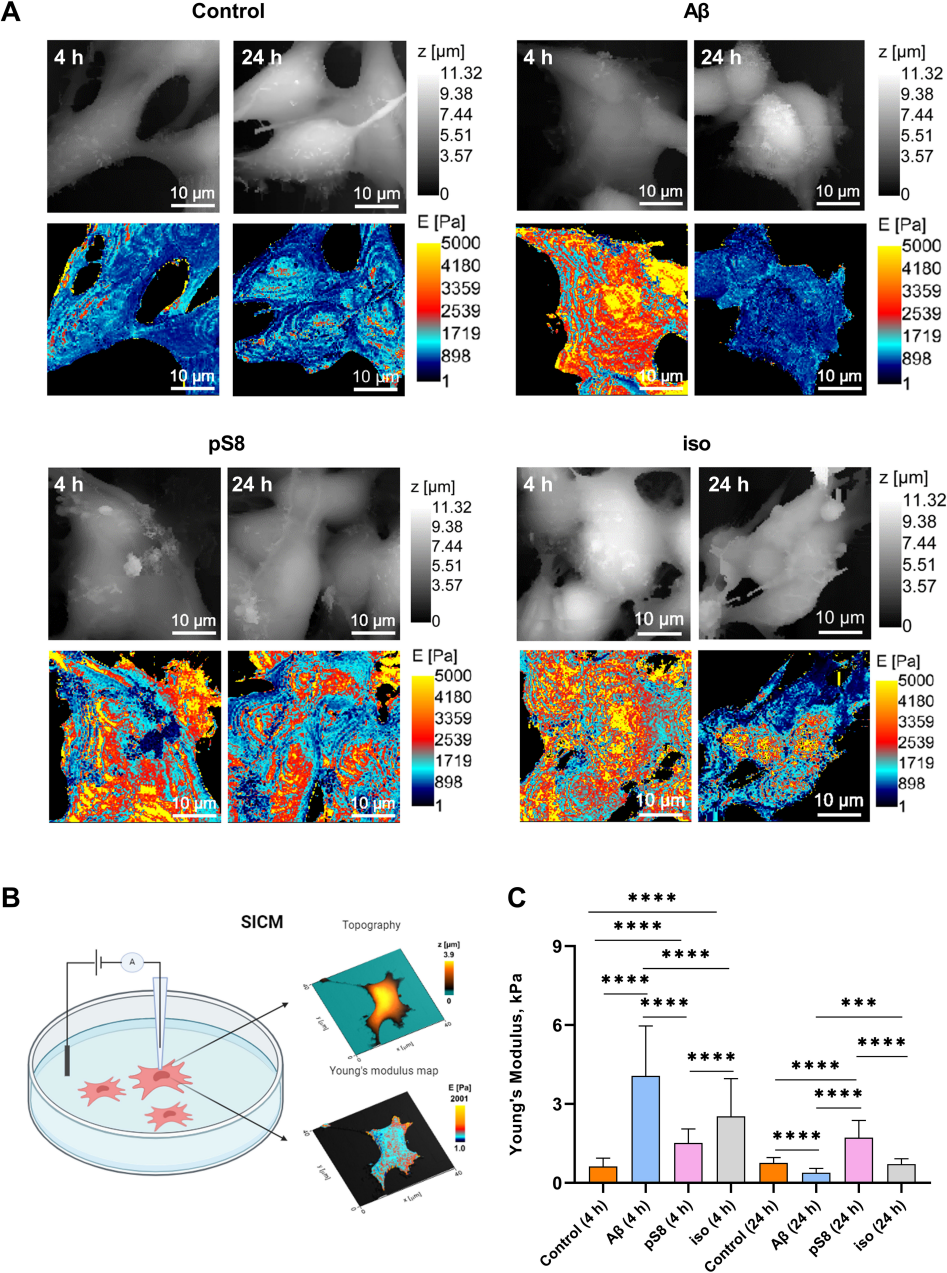
(A) Topography and Young’s modulus maps of SH-SY5Y cells incubated with different types of amyloids. Scale bar – 10 um. E – Young’s modulus, Pa. (B) Schematic representation of the process of topography and Young’s modulus measurements (C) Mean value ± sd of Young’s modulus of SH-SY5Y cells incubated with different types of Аβ. Number of cells in each point −40–100, ***p < 0,001, ****p < 0,0001.

**Figure 3:**
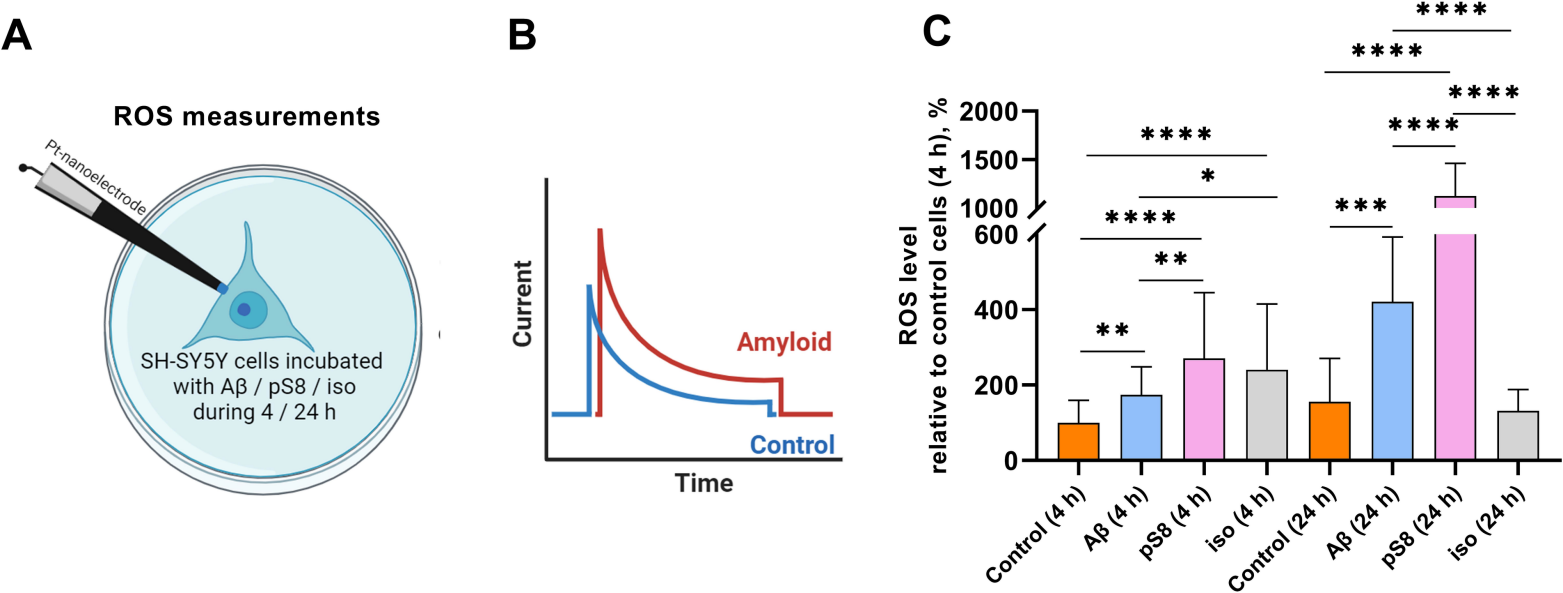
(A) Schematic representation of the process of intracellular ROS measuring. (B) Visualization of current recording inside a single SH-SY5Y cell. (C) The mean total ROS level ± sd of single SH-SY5Y cells incubated with different types of Аβ. Number of cells in each point −20–30, *p < 0,05, **p < 0,01, ***p < 0,001, ****p < 0,0001.

Cell stiffness is an important mechanical property and is measured as Young’s modulus or elastic modulus (Luo et al., 2016). When measuring the stiffness of SH-SY5Y cells using scanning ion-conductance microscopy (SICM), it was found that incubation with 10 μM Aβ_42_, pS8-Aβ_42_, iso-Aβ_42_ resulted in a significant increase in the Young’s modulus of the cells (Figure 2). After 4 h of incubation, Aβ_42_ had the greatest effect on cell stiffness, leading to a 6-fold increase in Young’s modulus compared to the control. However, after 24 h, Young’s modulus was significantly reduced for all Aβ isoforms except pS8-Aβ_42_. Aβ is known to induce oxidative stress in various cells (Butterfield, 2002; Kadowaki et al., 2005). When measuring the ROS level in neuroblastoma cells, the maximum increase in ROS was recorded after incubation with pS8-Aβ_42_, leading to a 3-fold and 7-fold increase in ROS levels after 4 and 24 h, respectively, compared to the control (Figure 3).

### 3.2 Beta-amyloid isoforms influence cofilin regulation

**Figure 4:**
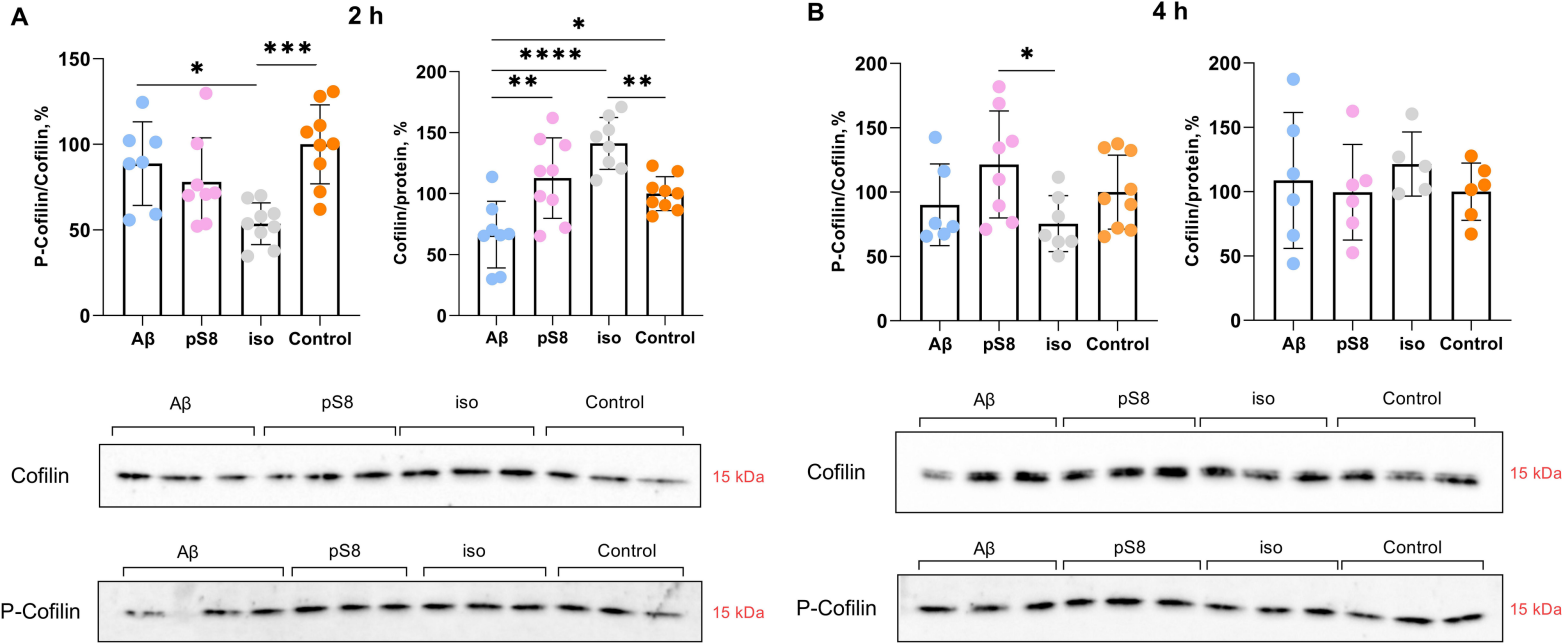
Effect of beta-amyloid isoforms (Аβ_42_, pS8-Аβ_42_, iso-Аβ_42_) at a concentration of 10 μM on cofilin phosphorylation in SH-SY5Y cells after 2 h (A) and 4 h (B) of incubation with peptides. The ratio of phosphorylated cofilin to total cofilin (P-cofilin/cofilin) and the ratio of total cofilin to total protein content in the sample (Cofilin/protein) was calculated for every sample and normalized by the corresponding value in the control sample. Photographs of membranes obtained after Western blot stained with antibodies to total or phosphorylated cofilin are presented. The number of values in each group *n* = 8–9 (А), *n* = 5–9 (В), *p < 0,05, **p < 0,01, ***p < 0,001, ****p < 0,0001.

Having observed the increase in cell stiffness using SICM, we then studied the signaling cascades by which Aβ can lead to changes in the mechanical properties of SH-SY5Y cells. It is known that the organization of actin filaments is considered the most important factor determining cell stiffness (Luo et al., 2016). Cofilin plays a significant role in the regulation of actin filament dynamics (Heredia et al., 2006). We measured the levels of total cofilin and phosphorylated (inactivated) cofilin after incubation with Aβ isoforms. After 30 min of incubation with beta-amyloid peptides, no changes in the ratio of phosphorylated cofilin to total cofilin (P-cofilin/cofilin) were detected (data not shown), but after 2 h, in the presence of iso-Aβ_42_, this ratio was reduced by 45% compared to the control and by 40% compared to Aβ_42_ (Figure 4A). Thus, incubation with iso-Aβ_42_ leads to dephosphorylation and activation of cofilin, which leads to actin depolymerization. It was also found that after 2 h of incubation, exposure to Aβ_42_ reduced cofilin expression by 35% compared to control, while exposure to iso-Aβ_42_ increased cofilin expression by 40% compared to control (Figure 4A). After 4 h, no effect of Aβ isoforms on the level of cofilin expression was detected, however, pS8-Aβ_42_ caused a 60% increase in the proportion of phosphorylated cofilin compared to iso-Aβ_42_ (Figure 4B).

**Figure 5:**
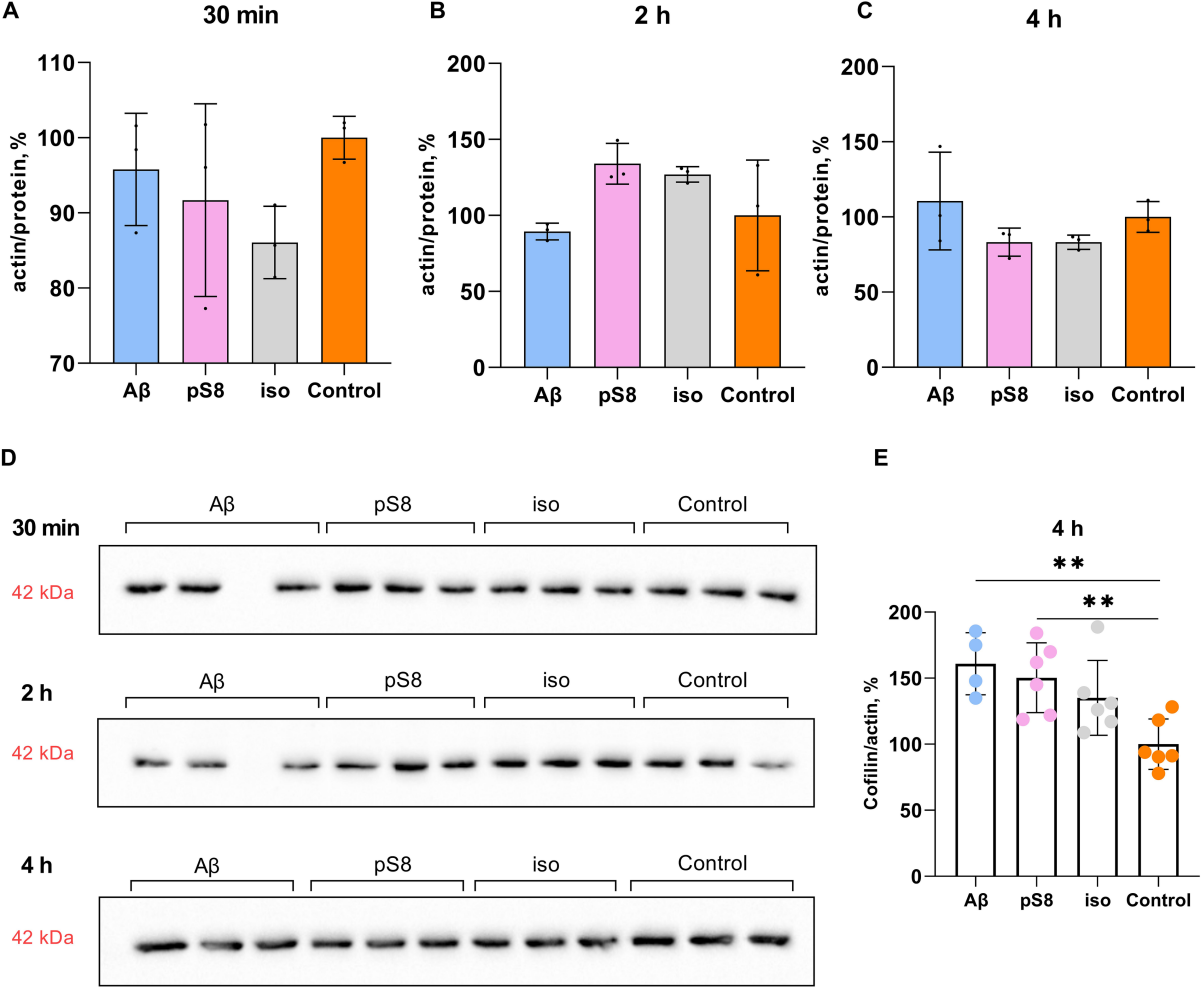
Effect of beta-amyloid isoforms (Аβ_42_, pS8-Аβ_42_, iso-Аβ_42_) at a concentration of 10 μM on actin levels in SH-SY5Y cells. The ratio of actin band intensity to total protein content in the sample cells after 30 min (A), 2 h (B), and 4 h (C) of incubation with peptides was calculated for every sample and normalized by the corresponding value in the control sample. (D) Western blot images of membranes stained with anti-beta-actin antibodies. (E) The ratio of cofilin to actin band intensities after 4 h of incubation with beta-amyloid peptides. The number of values in each group *n* = 3–6, **p < 0,01.

It is worth noting that Aβ isoforms do not lead to changes in actin expression levels neither at 30 min, 2 or 4 h of incubation (Figure 5). However, some studies suggest a dual role of cofilin in actin dynamics, indicating that actin filaments will be stabilized or destabilized depending on the ratio of cofilin to actin (Pavlov et al., 2007; Ohashi, 2015). Low concentration of cofilin promotes actin depolymerization, whereas high concentration of cofilin promotes actin nucleation and polymerization (Wang et al., 2020). When measuring the cofilin to actin ratio, it was found that after 4 h of incubation with Aβ_42_ and pS8-Aβ_42_, the cofilin/actin ratio increased by 60 and 50% compared to the control, respectively (Figure 5E). For iso-Aβ_42_, a tendency for this ratio to increase was also found.

### 3.3 Incubation with beta-amyloid isoforms leads to changes in the activity of kinases associated with the regulation of the actin cytoskeleton

**Figure 6:**
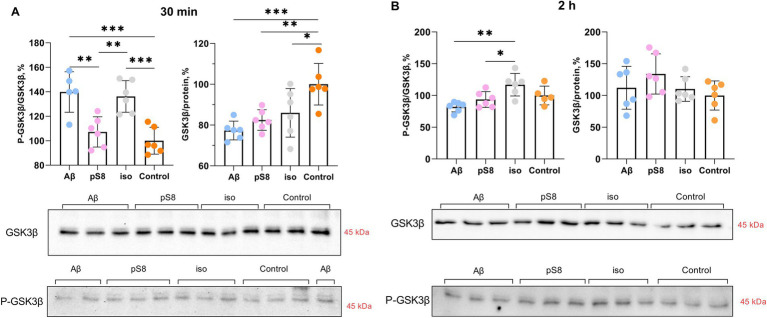
Effect of beta-amyloid isoforms (Аβ_42_, pS8-Аβ_42_, iso-Аβ_42_) at a concentration of 10 μM on GSK3β phosphorylation in SH-SY5Y cells after 30 min (A) and 2 h (B) of incubation with the peptides. The ratio of phosphorylated GSK3β to total GSK3β (P-GSK3β/GSK3β) and the ratio of total GSK3β to the total protein content in the sample (GSK3β/protein) was calculated for every sample and normalized by the corresponding value in the control sample. Photographs of membranes obtained after Western blot stained with antibodies to total or phosphorylated form of GSK3β are presented. The number of values in each group *n* = 5–6, *p < 0,05, **p < 0,01, ***p < 0,001.

Many protein kinases are involved in the regulation of cofilin, some of which are GSK3β, ERK1/2, p38 and LIMK1 (Huang et al., 2007; Mizuno, 2013; Bamburg et al., 2021) (Figure 1). GSK3β kinase phosphorylates tau and SSH phosphatase (Mizuno, 2013) and thus participates in the regulation of cytoskeletal dynamics in neuronal cells; phosphorylation of the kinase leads to its inactivation. It was found that Aβ_42_ and iso-Aβ_42_ caused an increase in the ratio of the phosphorylated form of GSK3β to total GSK3β (P-GSK3β/GSK3β) by 40% compared to the control after 30 min of incubation (Figure 6A). However, after 2 h of incubation with Aβ_42_, the P-GSK3β/GSK3β ratio significantly decreased and became 30% lower compared to iso-Aβ_42_ (Figure 6B). It is worth noting that this corresponds to a decrease in the phosphorylated form of cofilin after 2 h of incubation with iso-Aβ_42_ (Figure 4A): phosphorylation of GSK3β and its inactivation is accompanied by dephosphorylation and activation of SSH, which leads to dephosphorylation of cofilin (Figure 1). Thus, the early effect of Aβ_42_ leads to phosphorylation and, accordingly, inhibition of GSK3β, but then the reverse process occurs, leading to dephosphorylation of GSK3β and its activation. 30 min after the start of incubation with beta-amyloid peptides, the level of the total form of this kinase also decreases by 15–20% compared to the control. Importantly, the effects of Aβ_42_, pS8-Aβ_42_, and iso-Aβ_42_ on GSK3β phosphorylation differed, possibly indicating their different effects on the mechanical properties of SH-SY5Y. No changes in GSK3β phosphorylation were observed after 4 h of incubation with beta-amyloid peptides (data not shown).

**Figure 7:**
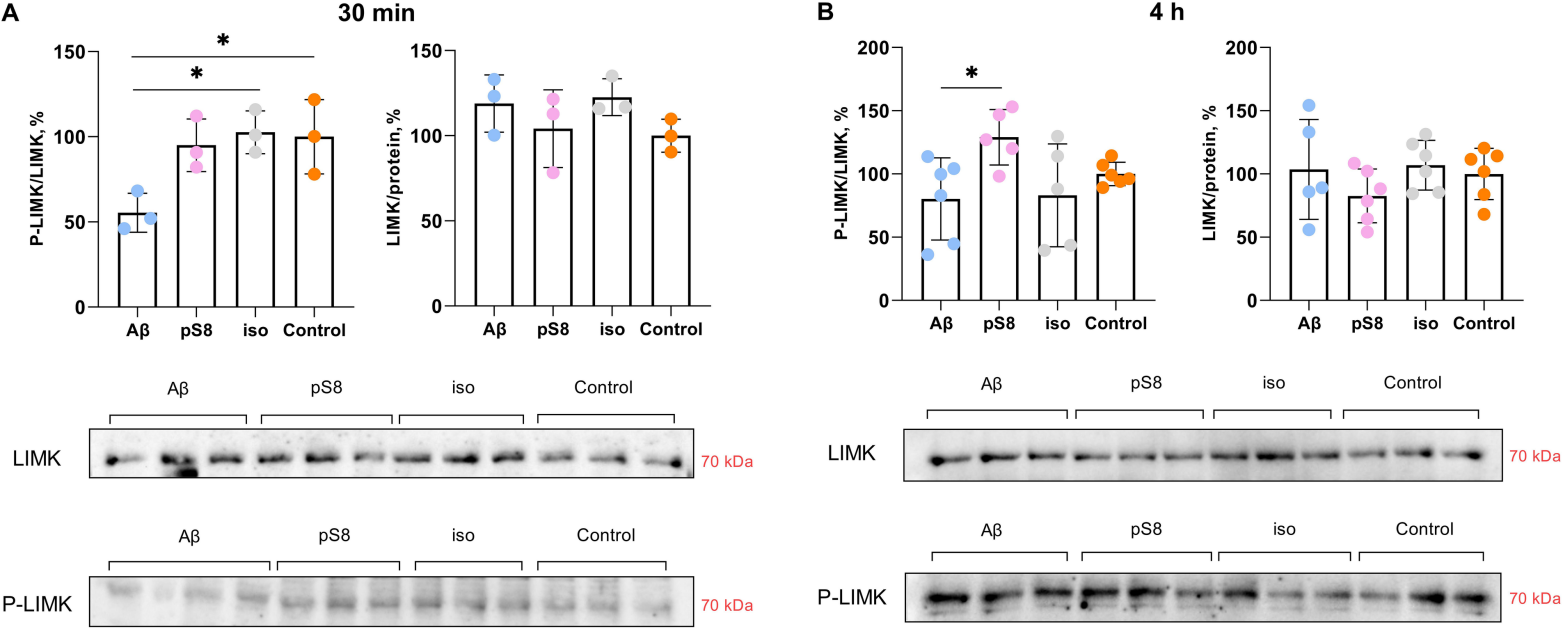
Effect of beta-amyloid isoforms (Аβ_42_, pS8-Аβ_42_, iso-Аβ_42_) at a concentration of 10 μM on LIMK phosphorylation in SH-SY5Y cells after 30 min (A) and 4 h (B) of incubation with peptides. The ratio of phosphorylated LIMK to total LIMK (P-LIMK/LIMK) and the ratio of total LIMK to the total protein content in the sample (LIMK/protein) was calculated for every sample and normalized by the corresponding value in the control sample. Photographs of membranes obtained after Western blot stained with antibodies to total or phosphorylated LIMK are presented. The number of values in each group *n* = 3 (А), *n* = 5–6 (В), *p < 0,05.

The kinases LIMK (Pelucchi et al., 2020) and ERK (Chatzifrangkeskou et al., 2018) phosphorylate cofilin, directly regulating its activity. Both kinases are activated by phosphorylation. Incubation with Aβ_42_ was found to alter LIMK phosphorylation: P-LIMK/LIMK ratio was reduced by 45% compared to the control after 30 min (Figure 7A). After 4 h of incubation with pS8-Aβ_42_, the P-LIMK/LIMK ratio increases by 60% compared to Aβ_42_ (Figure 7B). A tendency for this ratio to increase was also found for pS8-Aβ_42_ compared to control (Figure 7B).

**Figure 8:**
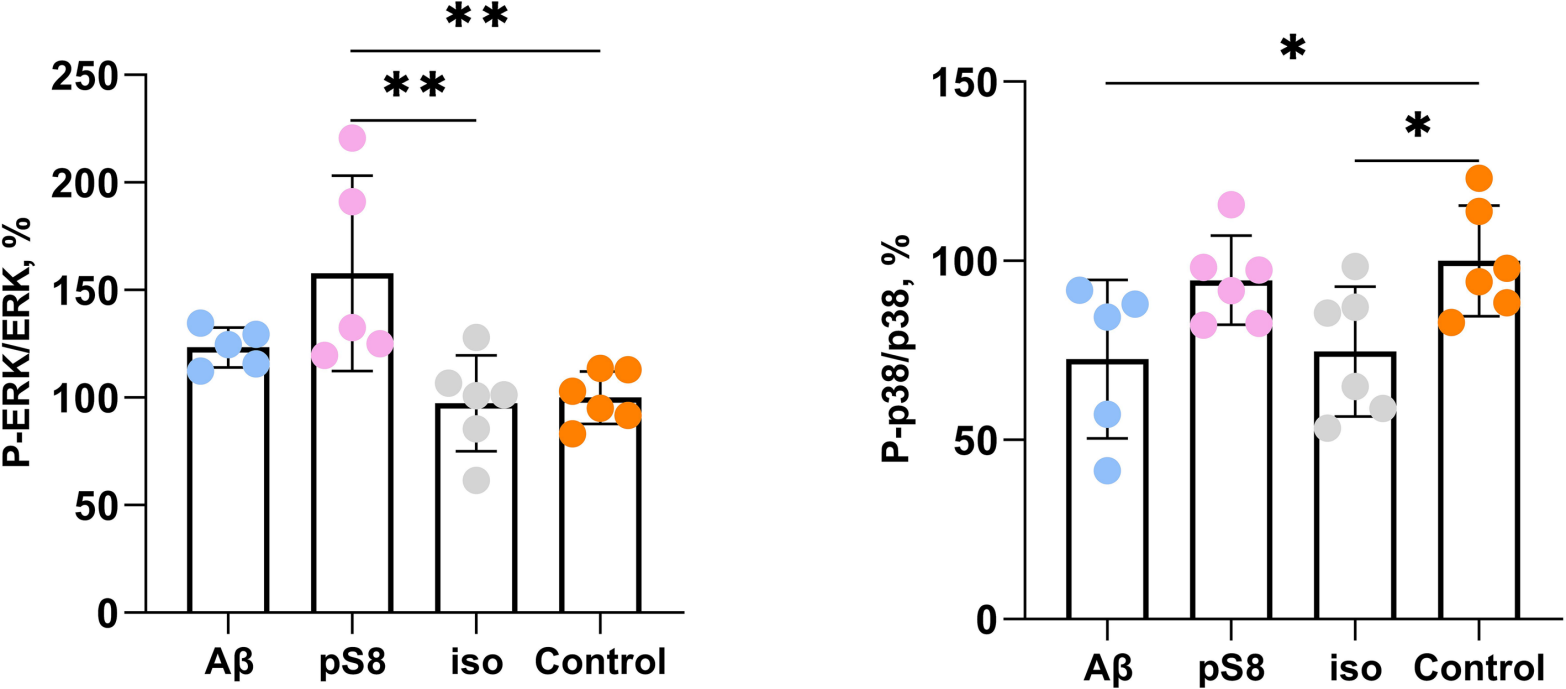
Effect of beta-amyloid isoforms (Аβ_42_, pS8-Аβ_42_, iso-Аβ_42_) at a concentration of 10 μM on phosphorylation of ERK and p38 in SH-SY5Y cells after 30 min of incubation with peptides. The ratios of the phosphorylated form to the total form calculated from the fluorescence intensity using Milliplex kits are presented. The number of values in each group *n* = 5–6, *p < 0,05; **p < 0,01.

We showed that incubation with 10 μM pS8-Aβ_42_ resulted in a 60% increase in the ratio of phosphorylated ERK to total ERK (P-ERK/ERK) after 30 min compared to the control (Figure 8). Thus, changes in ERK1/2 activity under the influence of pS8-Aβ_42_ can lead to changes in the cytoskeletal dynamics of SH-SY5Y cells.

The activity of p38 kinase, whose neuronal targets include intermediate filament, microtubule, and actin network proteins, was also assessed (Asih et al., 2020). P38 phosphorylates MAPK-activated protein kinase 2 (MK2) and subsequently activates LIMK, which in turn inhibits cofilin activity (Sugiura et al., 2009). It was found that exposure to Aβ_42_ and iso-Aβ_42_ reduces the degree of phosphorylation of this kinase after 30 min of incubation by 25–30% compared to the control (Figure 8). After 24 h of incubation with beta-amyloid peptides, no statistically significant activation of ERK and p38 was observed, which correlates with the fact the cell stiffness also reduces to control levels in case of 24 h Aβ_42_ and iso-Aβ_42_ incubation (Supplementary Figure S2). We also found that beta-amyloid isoforms lead to activation of the MAPK JNK after 30 min and 4 h of incubation (Supplementary Figure S3). However, no changes in the activation of NF-kB and Akt kinase were detected (Supplementary Figures S4, S5).

The obtained effects of beta-amyloid isoforms are summarized in Table 1.

**Table 1:** Effect of Aβ_42_, pS8-Aβ_42_, iso-Aβ_42_ on the dynamics of the actin cytoskeleton in SH-SY5Y cells.

Kinase/protein	Аβ	pS8	iso
GSK3β	Depolymerization (30 min) Polymerization (2 h)	Depolymerization (30 min)	Depolymerization (30 min)
Cofilin	Polymerization (2 h) Polymerization (4 h)	Polymerization (4 h)	Depolymerization (2 h) Polymerization (4 h)
LIMK	–	Polymerization (4 h)	–
Erk	–	Polymerization (30 min)	–
р38	Depolymerization (30 min)	–	Depolymerization (30 min)

The contribution of various kinases and cofilin to actin polymerization/depolymerization after 30 min, 2 and 4 h of incubation is shown.

## 4 Discussion

The mechanical properties of neuronal cells play a significant role in a wide range of biological functions, such as cellular homeostasis, proliferation, chemical and electrical signaling (Kolmogorov V. et al., 2023). These properties are determined mainly by cytoskeletal elements: actin cytoskeleton, microtubules, intermediate filaments (Luo et al., 2016). Oligomeric Aβ can alter cytoskeletal dynamics, which leads to changes in cell stiffness (Gao et al., 2019), and also stimulates ROS production in neuronal cells (Wang et al., 2010). In this work, the SICM method was used to measure the stiffness of SH-SY5Y cells in the presence of Aβ isoforms. Previously, using this method, it was established on SH-SY5Y cells that beta-amyloid leads to an increase in the Young’s modulus of the entire cell (Kolmogorov V. S. et al., 2023). We showed that Aβ_42_, pS8-Aβ_42_, iso-Aβ_42_ cause a significant increase in cell membrane stiffness, as well as an increase in the level of ROS (Figures 2, 3), which correlates with previously published data. The effects of beta-amyloid isoforms on these parameters differed: Aβ_42_ led to the greatest increase in stiffness after 4 h of incubation, and pS8-Aβ_42_ led to the greatest increase in stiffness and ROS production in cells after 24 h. At the same time, it is interesting that for Aβ_42_ and pS8-Aβ_42_, in contrast to iso-Aβ_42_, there is a tendency for the ROS level to increase over time.

The actin cytoskeleton is known to play a key role in synaptic transmission and plasticity (Pelucchi et al., 2020). Many studies have shown that disruption of the actin cytoskeleton using agents such as cytochalasin D, latrunculin A and latrunculin B results in a decrease in the Young’s modulus of cells (Roduit et al., 2009; Mihai et al., 2012; Pogoda et al., 2012; Louise et al., 2014; Ramos et al., 2014). It is worth noting that there is still no information on how much microtubules affect cell stiffness (Luo et al., 2016), and the possibility of intermediate filaments to influence stiffness independently of the rearrangement of actin filaments remains questionable (Gruenbaum and Aebi, 2014). Thus, the organization of actin filaments is considered to be the most important factor determining cell stiffness (Luo et al., 2016), so in our work we focused specifically on this element of the cytoskeleton.

Several actin-binding proteins are known to be altered in AD brains and animal models of AD (Pelucchi et al., 2020). Some of these important proteins are actin depolymerization factor (ADF) and cofilin. ADF/cofilin regulate actin filament dynamics (Heredia et al., 2006). ADF/cofilin promote actin depolymerization and create a new pool of G-actin monomers available for the formation of new filaments, thus increasing the rate of actin filament turnover in cells (Sarmiere and Bamburg, 2004; Bernstein and Bamburg, 2010; Rust, 2015). Since mammalian neurons contain approximately 5–10 times more cofilin than ADF (Minamide et al., 2000; Garvalov et al., 2007), we focused specifically on cofilin. Cofilin is inactivated by phosphorylation of Ser3 by LIM kinase 1 (LIMK1) and activated by dephosphorylation of Ser3 by Slingshot family protein phosphatases (SSH) (Pelucchi et al., 2020). We compared the effects of synthetic peptides Aβ_42_, pS8-Aβ_42_, and iso-Aβ_42_ on cofilin and its regulating kinases. We found that iso-Aβ_42_ activated cofilin by dephosphorylation, whereas Aβ_42_ and pS8-Aβ_42_ did not cause such changes. Aβ_40_ has previously been shown to increase the levels of Ser3-phosphorylated ADF/cofilin and Thr508-phosphorylated LIMK1 (P-LIMK1), which is accompanied by neuritic degeneration and neuronal cell death (Heredia et al., 2006). However, it is worth noting that this study used fibrillar Aβ_40_ and higher concentrations (20 μM), while soluble forms of Aβ_40_ did not change cofilin phosphorylation. Thus, it is possible that soluble Aβ_40_ and Aβ_42_ do not affect cofilin phosphorylation, in contrast to post-translationally modified forms of Aβ. At the same time, Kim et al. reported a decrease in cofilin phosphorylation in the brain of AD patients and in the forebrain of APP/PS1 mice, and also showed that the addition of Aβ_42_ oligomers to cortical neuronal cultures causes cofilin activation (Kim et al., 2013). These results are consistent with the activation of cofilin by iso-Aβ_42_ established in our work: isomerized Asp7 is contained in more than 50% of Aβ molecules of amyloid plaques and is also enriched in the soluble fraction (Bugrova et al., 2021; Mukherjee et al., 2021), which explains the activation of cofilin in the brain of patients with AD and *in vivo* models of AD. The activation of cofilin only by iso-Aβ_42_ may be one of the reasons for the greater toxicity of this isoform observed in many studies (Mitkevich et al., 2013). The researchers also note that the state of cofilin phosphorylation depends on both age and the stage of AD pathology (Barone et al., 2014). Interestingly, not only cofilin phosphorylation but also the cofilin to actin ratio influences actin filament stabilization. As the cofilin/actin ratio increases, cofilin triggers actin assembly and stabilizes the filaments (Andrianantoandro and Pollard, 2006; Bamburg and Bloom, 2009). We found that all beta-amyloid isoforms increased the cofilin/actin ratio after 4 h of incubation with the peptides, indicating polymerization of actin filaments and consistent with the increase in cell stiffness measured by SICM.

We also found differences in the effects of Aβ_42_, pS8-Aβ_42_ and iso-Aβ_42_ on the activity of cofilin-regulating kinases after 30 min of incubation: inhibition of GSK3β (Aβ_42_ and iso-Aβ_42_), inactivation of p38 (Aβ_42_ and iso-Aβ_42_). This leads to actin depolymerization, but the signaling pathways that cause this effect differ. Interestingly, pS8-Aβ_42_ appears to have a different signaling cascade via Erk activation, leading to actin polymerization. It is worth noting that this Aβ isoform had a distinctive pattern of effects on stiffness: after 4 h, pS8-Aβ_42_ caused a significantly smaller increase in the Young’s modulus of cells than Aβ_42_ and iso-Aβ_42_, but after 24 h, pS8-Aβ_42_ led to greater stiffness than the other isoforms. One of the reasons for these differences in Aβ isoforms may be the activation of different signaling cascades. It has been previously shown that some pathogenic properties of Aβ are neutralized by its phosphorylation (Jamasbi et al., 2017; Barykin et al., 2018). The activation of a different signaling pathway from other isoforms may be responsible for altered pathogenic properties of pS8-Aβ_42_. After 4 h, a change in signaling protein activation occurs, leading to actin polymerization and corresponding to an increase in cell stiffness measured by SICM. This discrepancy, at first glance, is explained by the fact that the rupture and depolymerization of actin filaments promotes an increase in the concentration of G-actin and the formation of free barbed ends of the filaments, which is necessary for the rapid reorganization of the actin cytoskeleton and further polymerization of actin (Mizuno, 2013; Pelucchi et al., 2020). It should be noted that the role of LIMK in cofilin regulation is ambiguous. Thus, it has been shown that Aβ_1–40_ and Aβ_25–35_ fibrils induce LIMK activation, which leads to cofilin inactivation (Heredia et al., 2006). However, Aβ_1–42_ was also found to activate LIMK1, which was paradoxically associated with increased cofilin activation, suggesting other pathways of cofilin regulation by Aβ (e.g., SSH1) (Wang et al., 2020; Kang and Woo, 2019). It has also been shown that cofilin activation is increased in AD with a simultaneous lack of changes in LIMK activation (Kim et al., 2013), which is consistent with our results.

It is known that ROS are not only damaging agents, but also mediators involved in cellular signaling and regulation (Corcoran and Cotter, 2013). ROS can affect cell stiffness in several ways (Supplementary Figure S6). In current study we focused on signaling cascades regulating actin cytoskeleton that can be induced by ROS. Thus, the activity of MAP kinases such as p38 and ERK can be regulated by ROS (Son et al., 2013; Zhang et al., 2016). Activation of p38 by ROS can affect the cytoskeleton both through cofilin (Figure 1) and through phosphorylation of the heat shock protein HSP27, which regulates microfilament dynamics, the redox state of actin, and some actin regulatory proteins (Guay et al., 1997, p. 27). At the same time, ERK, after stimulation by ROS, is able to activate actin regulatory complexes, which leads to actin polymerization (Taulet et al., 2012). Interestingly, pS8-Aβ_42_ caused the maximum increase in ROS observed in SH-SY5Y cells (Figure 3), while only pS8-Aβ_42_ led to ERK activation in our experiment (Figure 8). Hence, significant ERK activation by pS8-Aβ_42_ could be in part mediated by stimulated production of ROS. The researchers also note that oxidative stress activates GSK3β (Zhang et al., 2016), which plays an important role in both actin filament regulation and microtubule regulation. However, we did not observe these effects. It is known that ROS, through the oxidation of 14–3-3zeta, activate cofilin phosphatase, which leads to dephosphorylation and activation of cofilin (Kim et al., 2009). In addition to actin, ROS can also affect cell stiffness through other cytoskeletal elements. Thus, oxidative stress suppresses microtubule-associated proteins and affects tubulin through post-translational modifications. Neurofilaments become phosphorylated during oxidative stress, leading to the formation of protein aggregates (Gardiner et al., 2013). ROS are known to activate the MAP kinase JNK via several pathways (Zhang et al., 2016). We found that Aβ isoforms activate JNK (Supplementary Figure S3), which corresponds to increased ROS levels in SH-SY5Y cells (Figure 3). Signaling pathways such as NF-κB and PI3K-Akt also depend on ROS (Zhang et al., 2016), however, we did not detect activation of NF-κB factor and Akt kinase (Supplementary Figures S4, S5). Thus, beta-amyloid peptides used in our investigation do not activate the NF-κB and PI3K-Akt pathways in SH-SY5Y cells.

Reactive oxygen species are able to influence the reorganization of the actin cytoskeleton not only indirectly (via redox-sensitive enzymes), but also by direct oxidative modifications of actin. The researchers note that actin filaments can use oxidative stress for their reorganization in a context-dependent way, as oxidative stress can either increase cellular actin aggregation (Gardiner et al., 2013) or reduce the rate of filament formation via S-glutathionylation of actin (Haseena et al., 2022). In a mouse model of AD, a role of Aβ-induced oxidative stress in actin glutathionylation and F-actin reduction has been demonstrated (Kommaddi et al., 2019). It is also noted that deglutathionylation of G-actin leads to a 6-fold increase in the rate of polymerization (Wang et al., 2001). Thus, the effect of ROS on actin polymerization and, accordingly, cell stiffness is ambiguous and may depend on the cell type and additional regulatory mechanisms. Although depolymerization of actin filaments leads to a decrease in the Young’s modulus of cells, polymerization of actin filaments is not the only factor determining cell stiffness, and it is necessary to take the spatial organization of actin filaments into account (Luo et al., 2016).

## 5 Conclusion

In this work, it is shown that the effects of Aβ_42_, pS8-Aβ_42_ and iso-Aβ_42_ on signaling cascades associated with the regulation of mechanical properties of cells are different. This leads to the fact that, depending on the post-translational modification that the Aβ molecule acquires, beta-amyloid increases the Young’s modulus of the SH-SY5Y cell membrane and the level of ROS to varying degrees. Based on the obtained data, we proposed mechanisms mediating the effect of beta-amyloid peptide and its modified forms on the mechanical properties of neuronal cells. Thus, we showed that Aβ and its isoforms differently affect the activity of proteins that regulate the actin cytoskeleton, such as cofilin, GSK3*β*, LIMK, ERK and p38. We found that short-term incubation with beta-amyloid peptides activates signaling pathways that stimulate actin depolymerization, with the exception of ERK for pS8-Aβ_42_, while longer incubation results in activation of signaling cascades that lead to actin polymerization. In this case, pS8-Aβ_42_ has the most stable effect on cell stiffness. These results may explain the reasons for the different pathogenicity of Aβ_42_, pS8-Aβ_42_ and iso-Aβ_42_ for neuronal cells, which is important for a complete understanding of AD pathology.

## Data Availability

The original contributions presented in the study are included in the article/Supplementary material, further inquiries can be directed to the corresponding author.
