# Supplementary material for: Post-translational modifications of beta-amyloid modulate its effect on cell mechanical properties and influence cytoskeletal signaling cascades
Source: Front Mol Neurosci. 2024 Nov 14;17:1501874. doi: 10.3389/fnmol.2024.1501874 (PMC11602469; doi:10.3389/fnmol.2024.1501874)
Supplement: Supplementary file 1 [file Data_Sheet_1.pdf]

Supplementary Material

Post-translational modifications of beta-amyloid modulate its effect on cell mechanical properties and influence cytoskeletal signaling cascades

Kseniya B. Varshavskaya, Evgeny P. Barykin, Roman V. Timoshenko, Vasilii S. Kolmogorov, Alexander S. Erofeev, Petr V. Gorelkin, Vladimir A. Mitkevich1,* and Alexander A. Makarov

*** Correspondence:** Vladimir A. Mitkevich: mitkevich@gmail.com

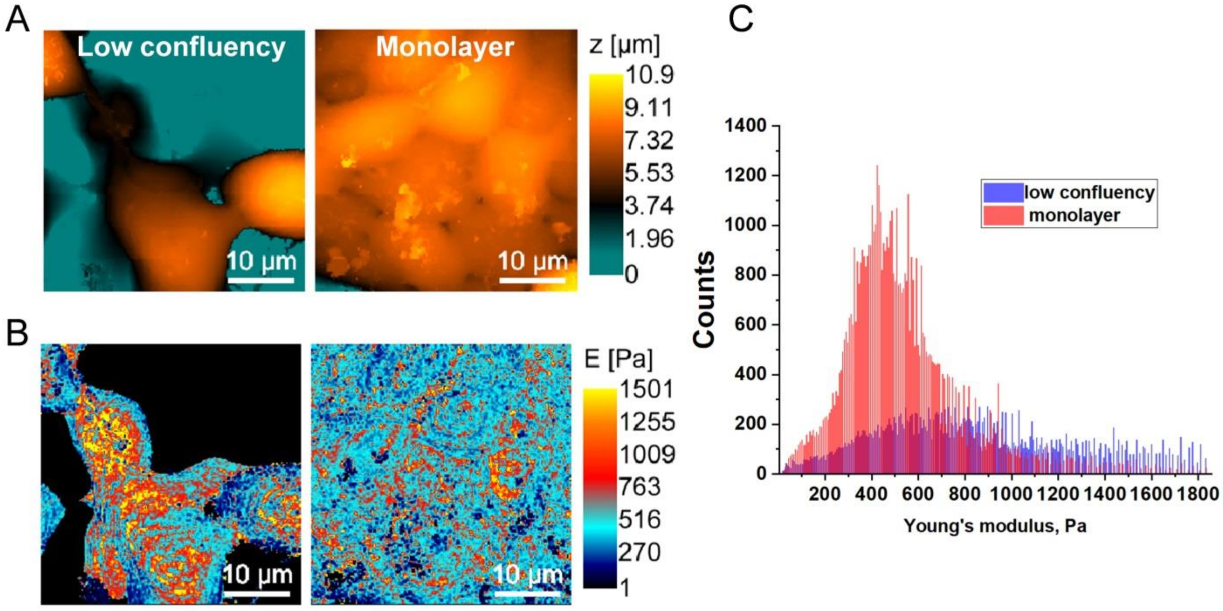

Supplementary Figure 1. Topography (A) and Young's modulus (B) maps of SH-SY5Y cells with different cell density; C – Young's modulus distribution.

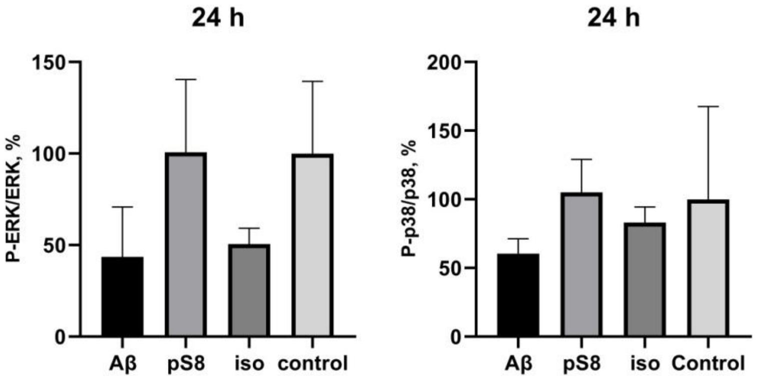

Supplementary Figure 2. Effect of beta-amyloid isoforms (Aβ_{42}, pS8-Aβ_{42}, iso-Aβ_{42}) at a concentration of 10 µM on phosphorylation of ERK and p38 in SH-SY5Y cells after 24 h of incubation with peptides. The ratios of the phosphorylated form to the total form calculated from the fluorescence intensity using Milliplex kits are presented.

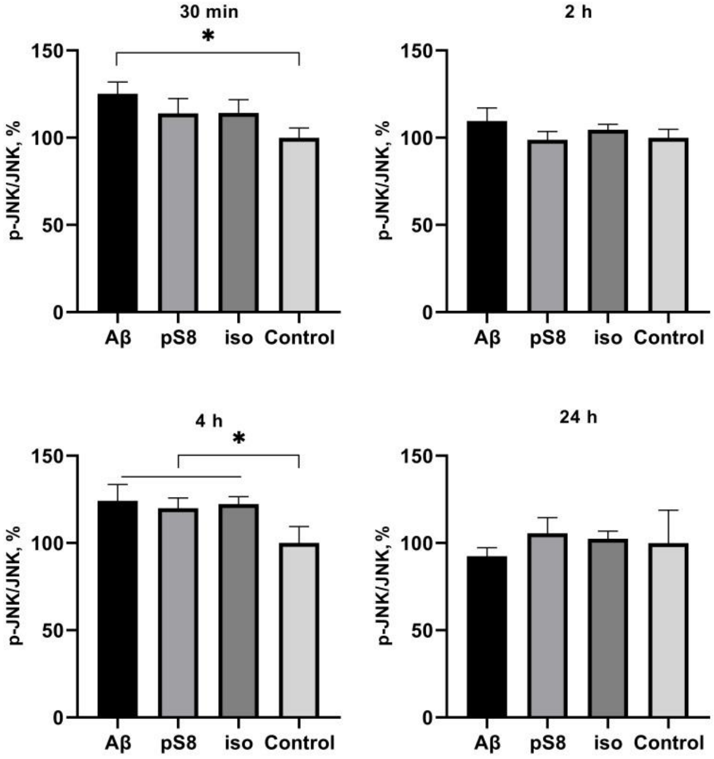

Supplementary Figure 3. Effect of beta-amyloid isoforms (Aβ_{42}, pS8-Aβ_{42}, iso-Aβ_{42}) at a concentration of 10 µM on phosphorylation of JNK in SH-SY5Y cells after 30 min, 2 h, 4 h and 24 h of incubation with peptides. The ratios of the phosphorylated form to the total form calculated from the fluorescence intensity using Milliplex kits are presented, * - $p < 0{,}05$.

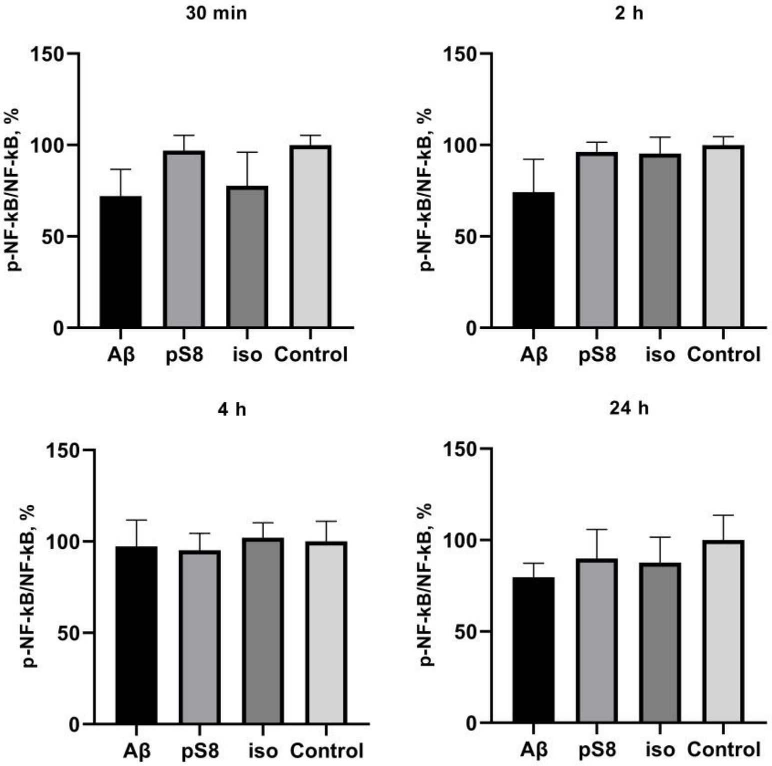

Supplementary Figure 4. Effect of beta-amyloid isoforms ($A\beta_{42}$, pS8-$A\beta_{42}$, iso-$A\beta_{42}$) at a concentration of 10 μM on phosphorylation of NF-kB in SH-SY5Y cells after 30 min, 2 h, 4 h and 24 h of incubation with peptides. The ratios of the phosphorylated form to the total form calculated from the fluorescence intensity using Milliplex kits are presented.

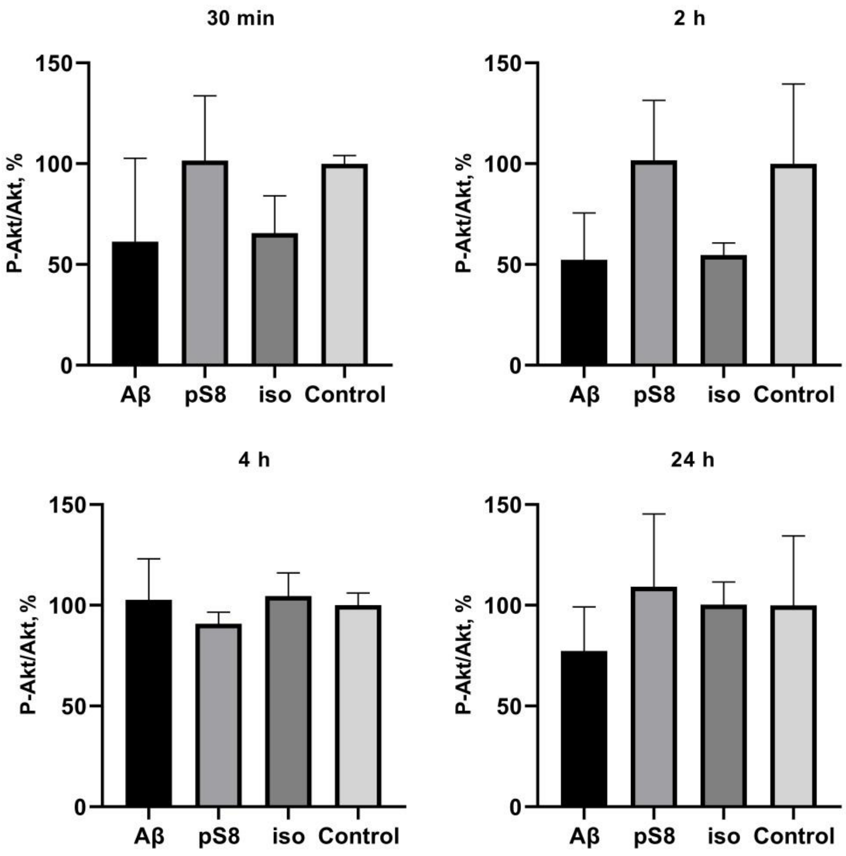

Supplementary Figure 5. Effect of beta-amyloid isoforms ($A\beta_{42}$, pS8-$A\beta_{42}$, iso-$A\beta_{42}$) at a concentration of 10 μM on phosphorylation of Akt in SH-SY5Y cells after 30 min, 2 h, 4 h and 24 h of incubation with peptides. The ratios of the phosphorylated form to the total form calculated from the fluorescence intensity using Milliplex kits are presented.

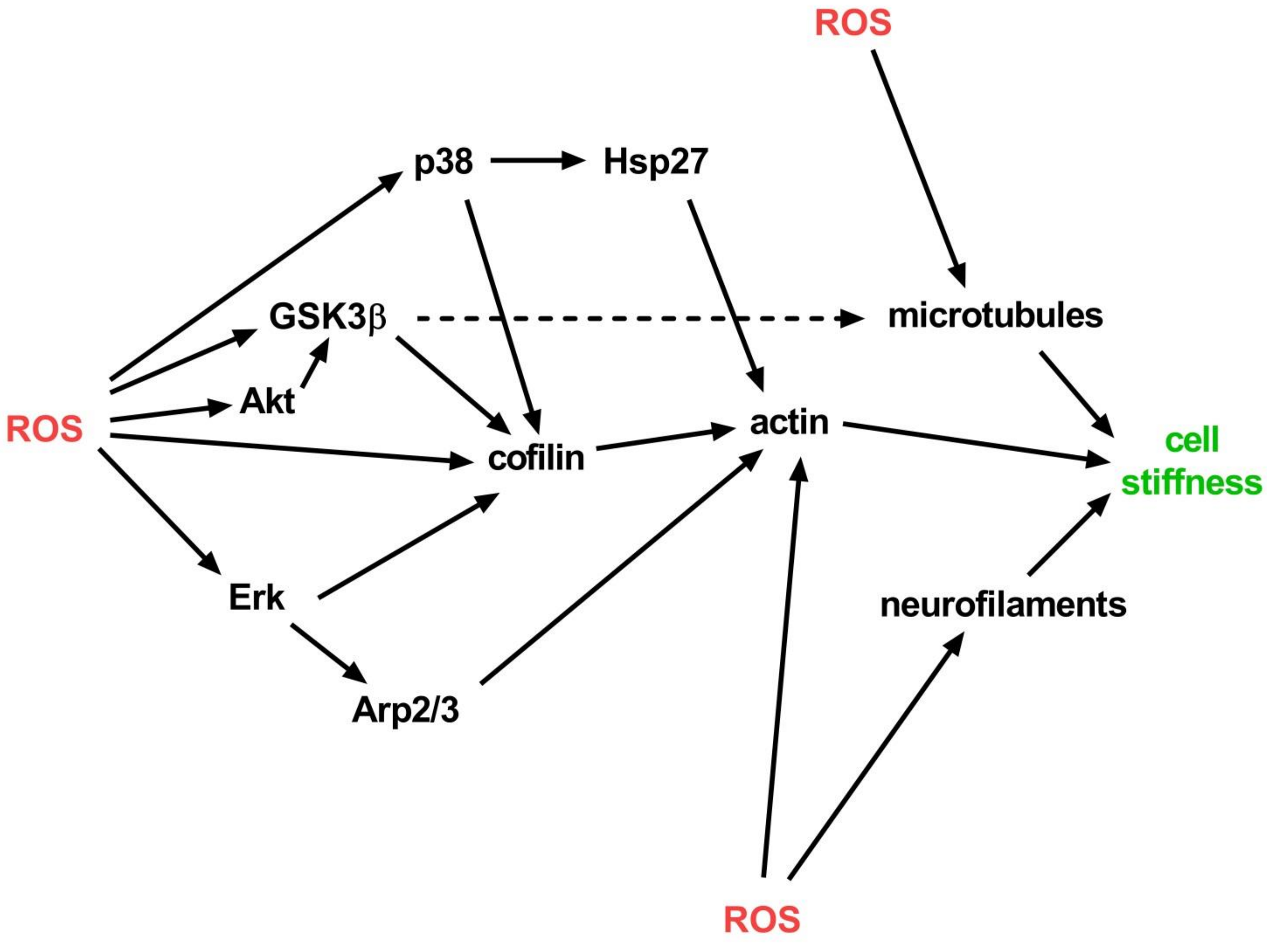

Supplementary Figure 6. Effect of ROS on neuronal cell stiffness. ROS can activate MAP kinases, such as p38 and ERK. Activation of p38 by ROS can affect the cytoskeleton both through cofilin and through phosphorylation of the heat shock protein HSP27. Phosphorylation of HSP27 prevents actin depolymerization and promotes stress fiber formation as an adaptive response to stress conditions. At ERK after stimulation with ROS is able to activate the WAVE2 regulatory complex, is required for activation activation of the Arp2/3 complex and subsequent actin polymerization. Oxidative stress activates GSK3β, which plays an important role in both actin filament regulation and microtubule regulation. ROS, through the oxidation of 14-3-3zeta, activate cofilin phosphatase, which leads to dephosphorylation and activation of cofilin, as well as to the formation of cofilin-actin rods. ROS activate Akt through effects on PTEN and phosphatases. ROS are also involved in oxidative modifications of actin: oxidative stress increases cellular actin aggregation, but on the other hand, oxidative stress promotes S-glutathionylation of actin, which reduces the rate of its polymerization. Oxidative stress suppresses microtubule-associated proteins and affects tubulin through post-translational modifications. Neurofilaments become phosphorylated during oxidative stress, leading to the formation of protein aggregates.

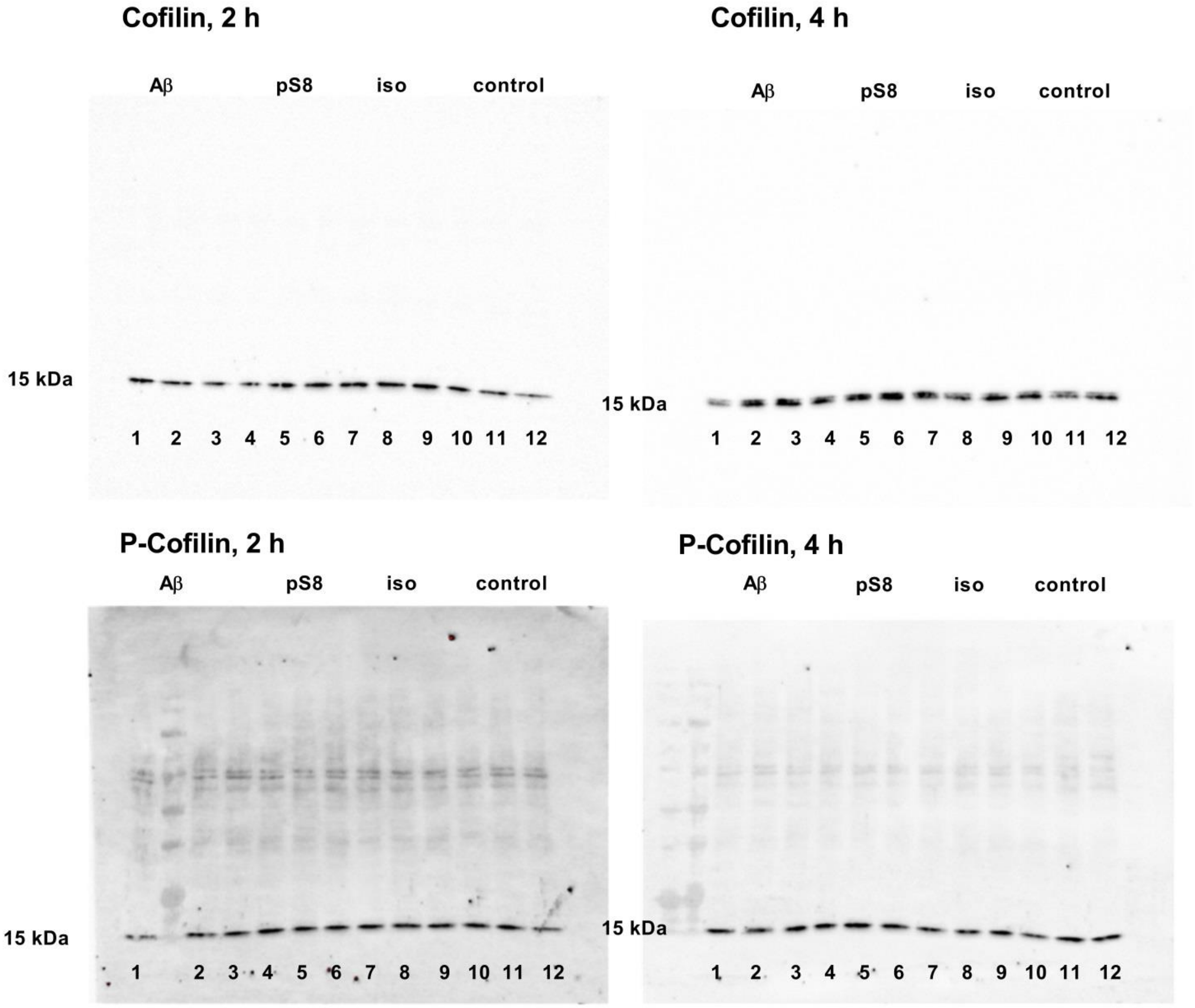

Supplementary Figure 7. Photographs of Western blot membranes stained with antibodies to total or phosphorylated cofilin. SH-SY5Y cells were incubated with 10 μM beta-amyloid isoforms (Aβ_{42}, pS8-Aβ_{42}, iso-Aβ_{42}) for 2 and 4 h. Lines 1-3 - A$\beta_{42,}$ lines 4-6 - pS8-Aβ_{42}, lines 7-9 - iso-Aβ_{42}, lines 10-12 – control (DMSO).

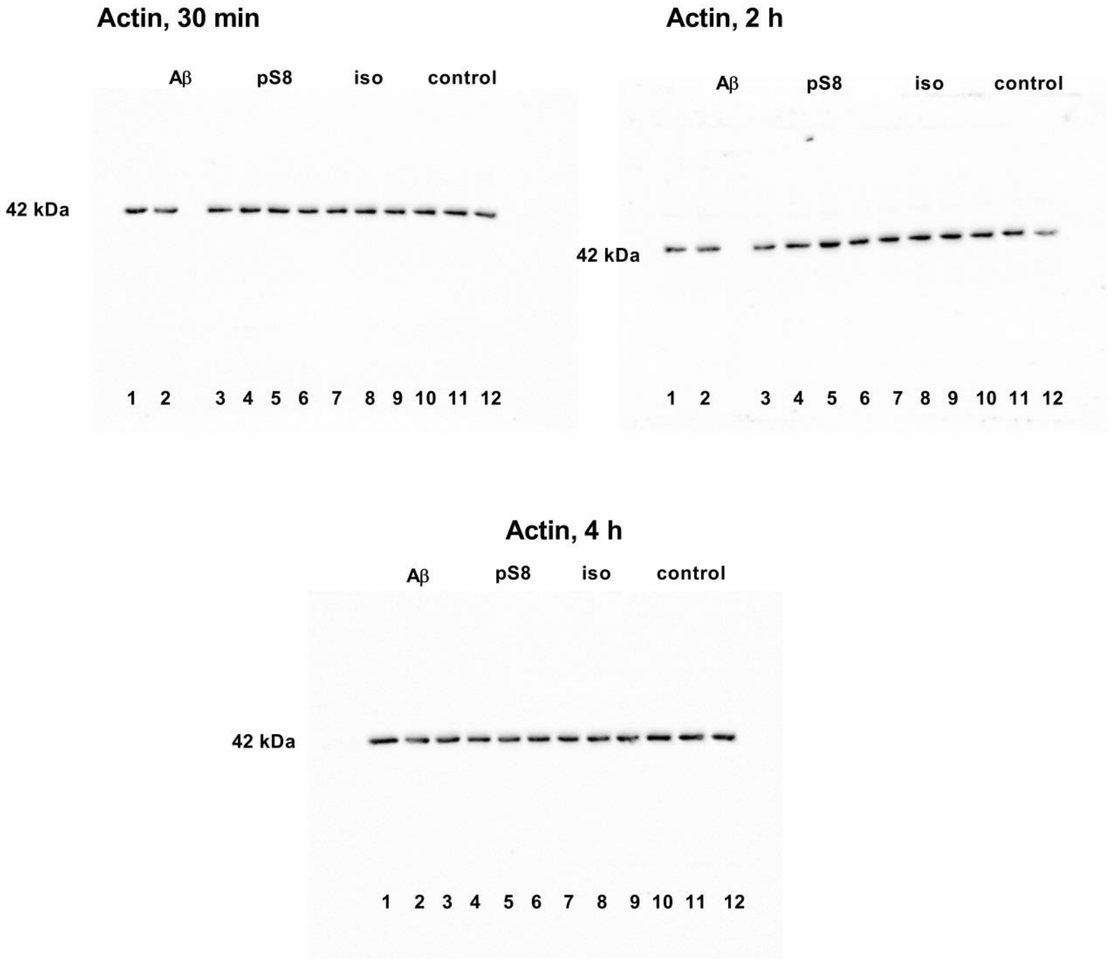

Supplementary Figure 8. Photographs of Western blot membranes stained with antibodies to beta-actin. SH-SY5Y cells were incubated with 10 μM beta-amyloid isoforms ($A\beta_{42}$, pS8-$A\beta_{42}$, iso-$A\beta_{42}$) for 30 min, 2 and 4 h. Lines 1-3 - $A\beta_{42,}$ lines 4-6 - pS8-$A\beta_{42}$, lines 7-9 - iso-$A\beta_{42}$, lines 10-12 – control (DMSO).

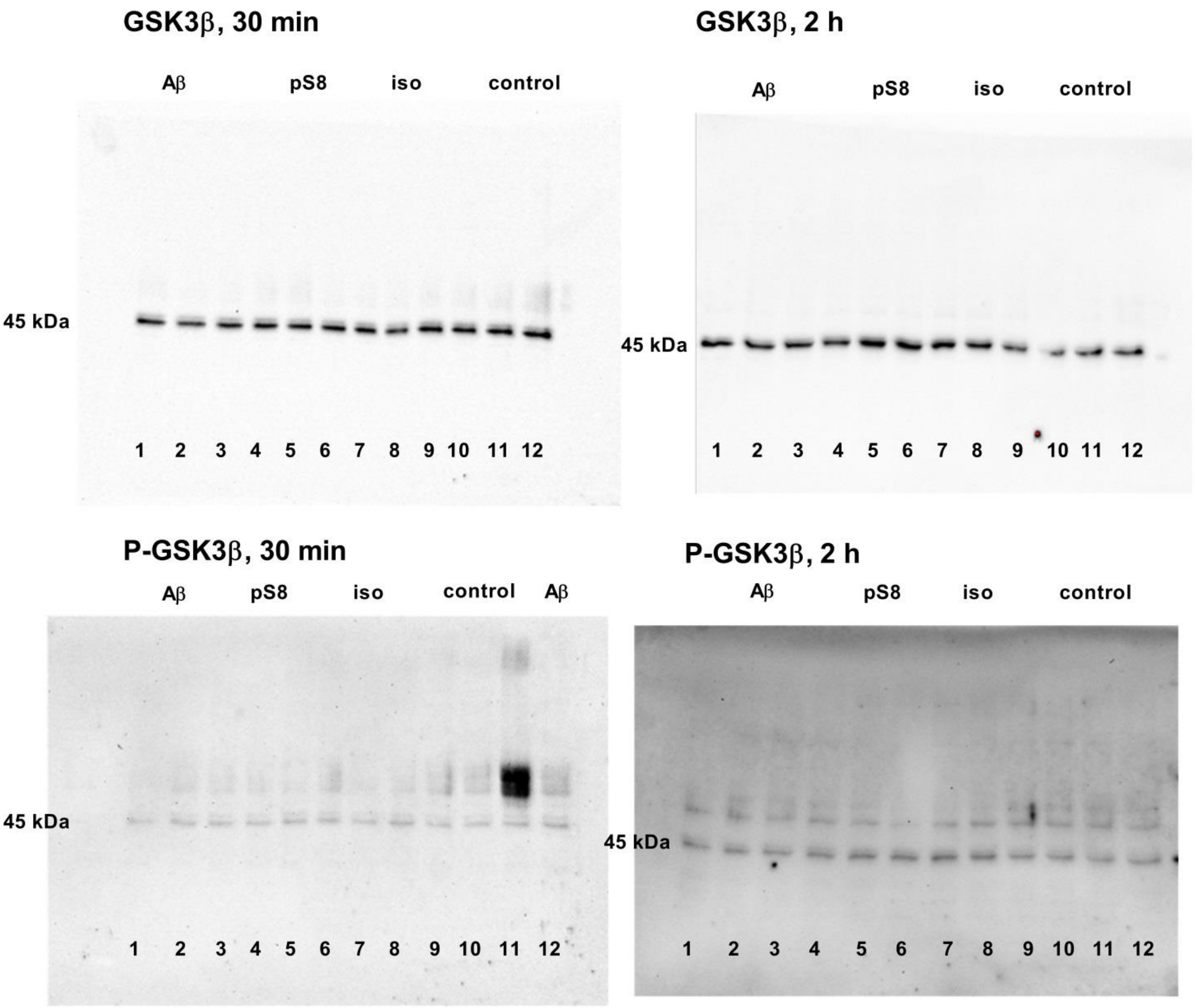

Supplementary Figure 9. Photographs of Western blot membranes stained with antibodies to total or phosphorylated GSK3β. SH-SY5Y cells were incubated with 10 μM beta-amyloid isoforms ($A\beta_{42}$, pS8-$A\beta_{42}$, iso-$A\beta_{42}$) for 30 min and 2 h. For GSK3β (30 min and 2 h) and P-GSK3β (2 h): Lines 1-3 - $A\beta_{42,}$ lines 4-6 - pS8-$A\beta_{42}$, lines 7-9 - iso-$A\beta_{42}$, lines 10-12 – control (DMSO). For P-GSK3β (30 min): Lines 1,2,12 - $A\beta_{42,}$ lines 3-5 - pS8-$A\beta_{42}$, lines 6-8 - iso-$A\beta_{42}$, lines 9-11 – control (DMSO).

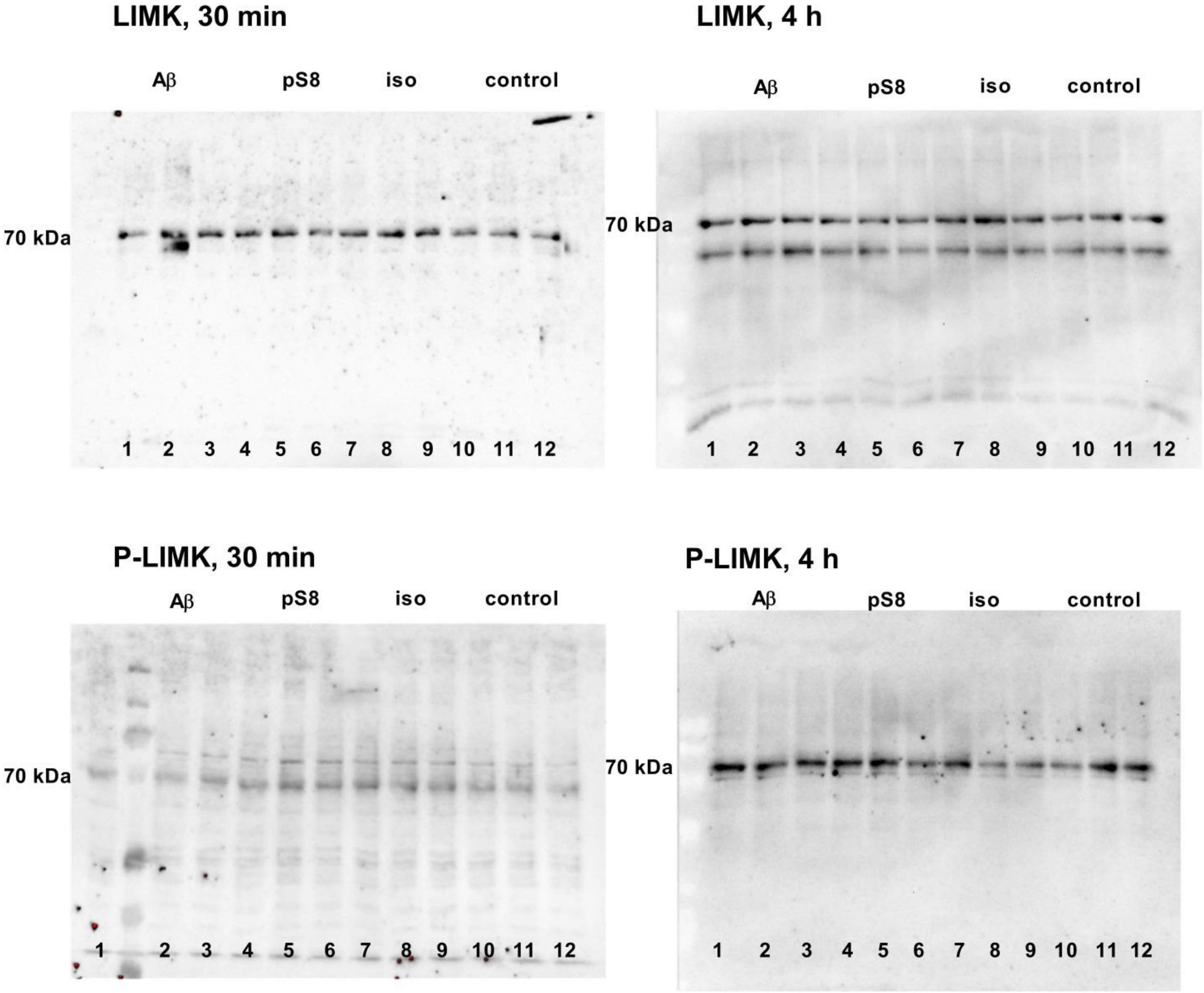

Supplementary Figure 10. Photographs of Western blot membranes stained with antibodies to total or phosphorylated LIMK. SH-SY5Y cells were incubated with 10 μM beta-amyloid isoforms ($A\beta_{42}$, pS8-$A\beta_{42}$, iso-$A\beta_{42}$) for 30 min and 2 h. Lines 1-3 - $A\beta_{42}$, lines 4-6 - pS8-$A\beta_{42}$, lines 7-9 - iso-$A\beta_{42}$, lines 10-12 – control (DMSO).
